# The Role of TRAIL Signaling in Cancer: Searching for New Therapeutic Strategies

**DOI:** 10.3390/biology13070521

**Published:** 2024-07-15

**Authors:** Cheng Luo, Shan He, Feng Shi, Jianhua Zhou, Li Shang

**Affiliations:** 1 Department of Pathology, National Clinical Research Center for Geriatric Disorders/Xiangya Hospital, Central South University, Changsha 410078, China; chengluo622@163.com (C.L.); zhoujh15@163.com (J.Z.); 2 Key Laboratory of Carcinogenesis and Cancer Invasion of Chinese Ministry of Education, Xiangya Hospital, Central South University, Changsha 410078, China; 13173817031@163.com (S.H.); shi_feng@csu.edu.cn (F.S.); 3 Key Laboratory of Carcinogenesis of National Health Commission, Cancer Research Institute and School of Basic Medical Science, Xiangya School of Medicine, Central South University, Changsha 410078, China

**Keywords:** tumor necrosis factor-related apoptosis-inducing ligand, cancer treatment, tumor microenvironment, apoptosis, immunotherapy

## Abstract

**Simple Summary:**

In the realm of modern medicine, the battle against cancer is a relentless pursuit that demands continuous innovation. Among the numerous elements in the complex world of cancer biology, TRAIL has emerged as a focal point for researchers and clinicians alike. Its diverse role in cancer, especially its influence on the tumor microenvironment (TME), has sparked extensive research. This article explores the intricacies of TRAIL’s function in cancer, revealing its dual nature—capable of both inhibiting and, conversely, promoting, cancer growth. TRAIL impacts a wide array of processes, from apoptosis to modulation of the immune response, and plays a crucial part in tumor initiation, progression, and invasion. Moving forward, the potential of TRAIL as a transformative element in cancer therapy is gaining recognition. New therapeutic approaches, including combination therapies, immunotherapies, and gene delivery methods, are being developed to leverage TRAIL’s potential for the benefit of cancer patients. Moreover, as our understanding of the TME and its effects on TRAIL’s efficacy grows, it is opening doors to more targeted and effective treatment strategies.

**Abstract:**

Cancer continues to pose a significant threat to global health, with its status as a leading cause of death remaining unchallenged. Within the realm of cancer research, the tumor necrosis factor-related apoptosis-inducing ligand (TRAIL) stands out as a critical player, having been identified in the 1990s as the tenth member of the TNF family. This review examines the pivotal role of TRAIL in cancer biology, focusing on its ability to induce apoptosis in malignant cells through both endogenous and exogenous pathways. We provide an in-depth analysis of TRAIL’s intracellular signaling and intercellular communication, underscoring its potential as a selective anticancer agent. Additionally, the review explores TRAIL’s capacity to reshape the tumor microenvironment, thereby influencing cancer progression and response to therapy. With an eye towards future developments, we discuss the prospects of harnessing TRAIL’s capabilities for the creation of tailored, precision-based cancer treatments, aiming to enhance efficacy and improve patient survival rates.

## 1. Introduction

Cancer remains a major challenge in contemporary medical research, requiring research into groundbreaking strategies that can shed light on the multifaceted processes of tumorigenesis and provide effective therapeutic interventions. Tumor necrosis factor-related apoptosis-inducing ligand (TRAIL) stands out among the large number of molecules being investigated for cancer therapy due to its ability to selectively induce cytotoxicity in malignant cells without harming healthy tissues [1]. As a member of the tumor necrosis factor (TNF) superfamily, TRAIL interacts with its death receptors DR4 and DR5 to trigger apoptosis, making it an attractive target for anticancer therapies [2].

However, despite its potential, barriers such as drug resistance and suboptimal pharmacokinetic profiles have hindered the widespread clinical use of TRAIL [3]. This review explores the critical role of TRAIL in tumorigenesis and progression. We focus on the dynamic interactions between TRAIL and the complex signaling networks that govern its apoptotic functions, as well as roles beyond these functions. In addition, we explore TRAIL-promoted intercellular communication between cancer cells and the tumor microenvironment, exploring extracellularly the current state of knowledge on the role of TRAIL and the mechanisms of resistance that have been identified.

In this review, we provide an overview of the status of ongoing clinical trials involving TRAIL and TRAIL-like drugs. It is worth pointing out that the utilization of the apoptotic pathway through gene delivery therapies targeting TRAIL offers a promising avenue for novel therapeutic approaches [4]. In this paper, we comprehensively review the role of TRAIL in carcinogenesis and evaluate the promise of targeting TRAIL signaling for cancer treatment. Our goal is to provide researchers with a clearer understanding of TRAIL therapeutic strategies for treating tumors and to inform clinical treatment.

## 2. TRAIL and Caspase-Dependent Apoptosis

### 2.1. TRAIL

**Figure 1:**
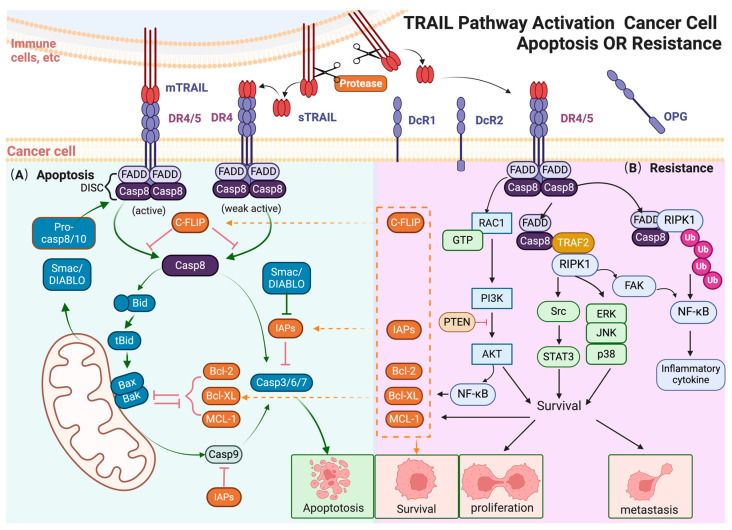
TRAIL pathway activation cancer cell apoptosis or resistance. (**A**) TRAIL induces exogenous and endogenous apoptotic signals in sensitive cancer cells. In the exogenous pathway, mTRAIL binds to DR4/5, leading to the formation of the DISC, activation of caspases, and eventually cell apoptosis; in the endogenous pathway, TRAIL-activated caspase-8 cleaves Bid, triggering mitochondrial membrane disruption and cytochrome c release. This leads to apoptotic body formation, activating procaspase-9 and downstream caspases, resulting in cell apoptosis. (**B**) Non-apoptotic signals induced by TRAIL in apoptosis-resistant cancer cells. In TRAIL-insensitive cells, TRAIL can activate various protein kinases, including NF-κB, PI3K/AKT, JNK, p38, ERK, and STAT3, leading to pro-tumorigenic responses. These pathways promote cell proliferation, invasion, and metastasis. NF-κB activation, mediated by the FADD-RIPK1 “FADDosome” complex, leads to the transcription of anti-apoptotic genes. The MAPK family, particularly ERK, JNK, and p38, contributes to TRAIL-induced non-apoptotic effects, supporting apoptotic resistance and promoting cell survival. AKT activation upregulates anti-apoptotic factors and stimulates NF-κB, contributing to resistance. Other signaling molecules like RIPK1, Src, and STAT3 also enhance pro-survival, migration, and invasion outcomes. The arrow and t-bar represent activated and inhibitory interactions respectively. (Figure was created with Biorender.com, accessed on 1 June 2024).

The apoptotic ligand TRAIL (TNFSF10), the tenth member of the tumor necrosis factor (TNF) superfamily, is a type II transmembrane protein [2]. TRAIL plays a pivotal role in apoptosis induction (Figure 1) [5,6]. There are two main forms of TRAIL: membrane-bound TRAIL (mTRAIL) and soluble TRAIL (sTRAIL) [2]. mTRAIL, consisting of 281 amino acids, is expressed in various tissues and cell types, with a predominant presence on the cell surface of immune cells. The extracellular C-terminal region of TRAIL can be proteolytically cleaved from the cell surface by cysteine proteases, namely sTRAIL [7]. In healthy adult plasma, sTRAIL is typically found at a concentration of about 100 pg/mL, and at this concentration, sTRAIL could not induce apoptosis in most cell lines [8]. Moreover, a naturally occurring splice variant of TRAIL, called TRAILshort, has been identified as a membrane-bound short form of TRAIL, which lacks the apoptosis-inducing activity [9,10,11]. Monoclonal antibodies (mAbs) selectively targeting TRAILshort have been shown to enhance cancer susceptibility to TRAIL and improve the efficacy of autologous CD8+ T cells in isolated primary tumors [11].

### 2.2. Receptors of TRAIL

Human TRAIL can interact with five receptors. Among these, death receptor 4 (DR4/TRAIL-R1, encoded by TNFRSF10A) and death receptor 5 (DR5/TRAIL-R2, encoded by TNFRSF10B) are regarded as pro-apoptotic receptors [12]. They both feature the same death domain (DD) motif, which enables the recruitment of apoptotic signaling molecules to initiate cell death. Both DR4/5 could be activated by mTRAIL, while sTRAIL is only able to induce the trimerization of DR4 [12]. The molecular aggregation capacity of mTRAIL is 100–1000 times stronger than that of sTRAIL, which may explain why sTRAIL can only activate DR4 and exhibits significantly lower cytotoxic potential [12]. Because of the lack of DD motif, decoy receptor 1 (DcR1/TRAIL-R3, encoded by TNFRSF10C), decoy receptor 2 (DcR2/TRAIL-R4, encoded by TNFRSF10D) and osteoprotegerin receptor (OPG) cannot induce apoptosis upon TRAIL binding [2,13]. In contrast, they may compete with the pro-apoptotic receptors DR4 and DR5 for TRAIL binding, and thereby inhibit TRAIL-mediated apoptosis [13]. It is worth noting that OPG is a soluble receptor, which acts as the decoy receptor for TRAIL [14,15]. In a physiological environment, the affinity of TRAIL for OPG is significantly weaker than its affinity for membrane-bound receptors, suggesting that OPG’s role in influencing TRAIL signaling is relatively minor [13].

TRAIL receptors are most often found on the cell membrane surface, but are also found in the cytoplasm and nucleus. The subcellular localization affects the function of TRAIL receptors. Studies have shown that nuclear expression of DR5 inhibits the maturation of let-7 and increases tumor cell proliferation, which is independent of the interaction between DR5 and TRAIL [16,17]. Moreover, post-translational modification is involved in the activation of TRAIL receptors [18]. Studies have indicated that O-glycosylation of DR5 can regulate the sensitivity of cells to TRAIL-induced apoptosis [19], while N-glycosylation of DR4 can enhance its pro-apoptotic effect [20].

### 2.3. Canonical Signaling Mediated by TRAIL

TRAIL coordinates a complex series of molecular interactions in the classical exogenous and endogenous apoptotic pathways that ultimately lead to the programmed death of malignant cells (Figure 1A). TRAIL has the capability to initiate the exogenous apoptotic pathway. mTRAIL induces the trimerization of DR4/5 receptors on targeted cells, which is necessary for the recruitment of adaptor proteins, such as Fas-associated death domain (FADD). Procaspase-8 and pro-caspase-10 then combine with FADD to form the DISC complex [21]. The DISC complex integrates extracellular signals and triggers the apoptotic cascade, including the cleavage of caspase-8/10 and the activation of downstream effectors such as caspase-3/6/7 [22].

In addition, TRAIL also plays an important part in the endogenous apoptotic pathway. TRAIL-activated caspase-8 also cleaves BH3 interaction domain death agonist (Bid) into the carboxy-terminal fragment of Bid (tBid). tBid promotes the oligomeric polymerization of Bax and Bak on the mitochondrial outer membrane, which leads to the mitochondrial membrane potential depolarization and the release of cytochrome c [23]. Cytochrome c recruits apoptotic peptidase activator 1 (Apaf-1) and procaspase-9 to form apoptotic bodies, which activates caspase-9 and thereby induces endogenous apoptosis [24].

## 3. The Multiple Roles of TRAIL in the Tumor Microenvironment

**Figure 2:**
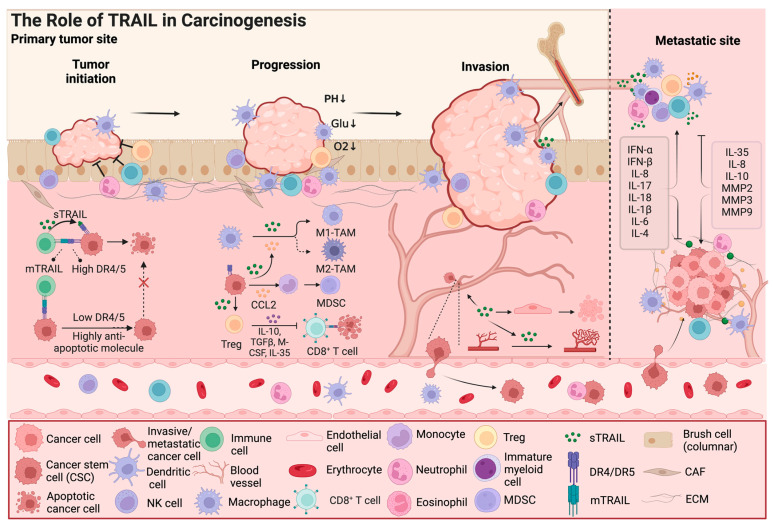
The role of TRAIL in carcinogenesis. In addition to its effect on cancer cell apoptosis, TRAIL also exhibits dual effects on components of the tumor microenvironment, such as cancer stem cells and immune cells; Furthermore, TRAIL signaling is involved in cancer progression by regulating the tumor microenvironment, including remodeling of blood vessels and invasion and metastasis of cancer cells. TAMs, tumor-associated macrophages; MDSCs, myeloid-derived suppressor cells; CAFs, cancer-associated fibroblasts; CCL2, C-C Chemokine Ligand 2; IL, Interleukin; IFN, Interferon; TGF, tumor growth factor; M-CSF, macrophage colony stimulating factor; MMP, matrix metalloproteinase; ECM, extracellular matrix. The arrow and t-bar represent activated and inhibitory interactions, respectively. (Figure was created with Biorender.com, accessed on 1 June 2024).

Although TRAIL can induce apoptosis in various cancer cell lines, many other cell lines and most primary cancers are TRAIL resistant [3]. In TRAIL-resistant cancer cells, TRAIL can activate cell survival signal pathways, including IkB/nuclear factor κB (NF-κB), PI3K/AKT (phosphoinositide 3-kinase/protein kinase B), c-Jun N-terminal kinase (JNK), p38, extracellular signal-regulated kinase (ERK), and SRC-signal transduction and transcriptional activator 3 (STAT3) pathways (Figure 1B). In addition to cancer cells, TRAIL also exhibits dual effects on the components of the tumor microenvironment, such as cancer stem cells and immune cells [25]. In the following section, we mainly discuss how the TRAIL signal participates in cancer progression by regulating the tumor microenvironment (Figure 2).

### 3.1. Cancer Stem Cells Are TRAIL Resistant

Cancer stem cells (CSCs) are a subpopulation of cancer cells with self-renewal capacity and multi-lineage differentiation potential which can drive tumor initiation [26,27,28]. CSCs can be identified in most prevalent cancers, as they express various typical cellular markers, such as CD133, CD44, and ALDH [29,30]. Studies have shown that CSCs with high death receptor expression in breast and colon cancer respond effectively to TRAIL treatment [31,32]. Interestingly, an increasing body of evidence suggests that CSCs often exhibit low levels of DR4/5 and high levels of anti-apoptotic molecules, rendering them highly resistant to apoptosis [33]. These anti-apoptotic molecules (c-FLIP, IAPs, and Bcl-2, among others) act as blockers in the pathway of TRAIL-induced apoptosis (Figure 1B).

In addition, targeting TRAIL signals has also become a strategy to solve TRAIL-resistant CSCs. Studies have shown that the inhibitor of EZH2 sensitizes colon CSCs for TRAIL-induced apoptosis by upregulating DR4/5 expression [34]. The anti-apoptotic and pro-survival signaling pathways are also responsible for TRAIL resistance. The CSC surface marker CD133 can inhibit TRAIL-induced apoptosis by upregulating FLICE-like inhibitory protein (FLIP) expression and activating the PI3K-AKT signal [35]. The tumor suppressor PTEN, which inhibits the carcinogenic PI3K/AKT signaling pathway, can be negatively regulated by miR-25 overexpression, thus enhancing the presence of TRAIL-resistant CSCs [36]. Inhibition of JNK signaling can reduce DcR1 expression and increase DR4/5 expression through an IL-8-dependent autocrine process in pancreatic CSCs, which enhances the susceptibility to TRAIL treatment [37]. Overexpression of β-catenin and GLI3 in CSCs could impair TRAIL-induced apoptosis by activating the Wnt and Hedgehog signaling pathways [38,39].

### 3.2. TRAIL Has Dual Effects on Immune Cells

TRAIL is expressed in macrophages, cytotoxic T cells, neutrophils, natural killer (NK) cells, and dendritic cells, which plays a crucial role in antitumor immune responses [17]. TRAIL acts as a crucial element in inducing apoptosis of tumor cells particularly secreted by cytotoxic T cells and NK cells [40]. TRAIL can promote the polarization of human macrophages towards the pro-inflammatory/antitumor M1 phenotype, which reinforce the cytotoxic effect on malignant cells [41,42]. TRAIL also has the ability to induce the apoptosis of myeloid-derived suppressor cells (MDSCs), which enhances the antitumor activity of the immune system [43]. Additionally, TRAIL can trigger the production of pro-inflammatory cytokines, including IL-1β, IL-6, and TNF-α, which amplify the inflammatory response against tumors [41].

Conversely, TRAIL may contribute to an immunosuppressive microenvironment. IL-35 can induce N2 neutrophil polarization and promote neutrophil infiltration into the tumor microenvironment, which augments the proangiogenic and immunosuppressive function of neutrophils [44]. IL-10/IL-35 double-deficient Treg cells typically rely on the enhancement of TRAIL expression and the release of soluble TRAIL to provide functional plasticity [45].

Regulatory T (Treg) cells have a critical role in the prevention of autoimmunity, which also suppress robust antitumor immune responses. High doses of TRAIL have been demonstrated to boost the proliferation of Tregs, and also enhance the recruitment and polarization of immunosuppressive M2-like macrophages [46,47]. TRAIL-triggered CCL2 secretion from TRAIL-resistant cancer cells can drive monocyte polarization to MDSCs and M2-like macrophages, which promotes accumulation of tumor-supportive immune cells in the tumor microenvironment [48]. In a murine cholangiocarcinoma model, TRAIL from cancer cells is reported to facilitate MDSC proliferation though the noncanonical TRAIL signaling with consequent NF-κB activation [49]. TRAIL-induced immune tolerance may be one reason for immune escape of cancer cells which sustains tumor growth and metastasis.

### 3.3. The Interaction of the TRAIL Signal Pathway and Other Cytokines Influencing the Tumor Microenvironment

Cytokines regulate the TRAIL signal and TRAIL-induced apoptosis. Type I interferon (IFN) secreted by immune cells, such as DC and NK cells, can enhance TRAIL expression in neutrophils and NK cells [50,51]. Pro-inflammatory cytokines, such as IL-8, IL-17, and IL-18, have been shown to promote the release of TRAIL from human neutrophils and NK cells [52,53]. IL-1β can induce TRAIL expression in human umbilical cord mesenchymal stem cells (hUCMSCs), which induces the apoptosis of breast cancer cells [54]. Other studies revealed that IL-1β can also increase the expression of OPG, the decoy receptor of TRAIL, which protects cancer cells from TRAIL-induced apoptosis [55]. IL-6 is reported to enhance TRAIL-induced apoptosis through p53-dependent upregulation of DR4 and DR5 [56]. Similarly, IL-4 treatment can significantly upregulate DR4 expression [57]. IL-35, an immunosuppressive cytokine, has been shown to downregulate TRAIL expression in neutrophils [44].

The TRAIL signal may stimulate the release of cytokines. In non-small cell lung cancer and colon cancer cells, TRAIL receptor signaling could stimulate the secretion of IL-8 constitutively, which contributes to a pro-tumorigenic microenvironment [58,59]. TRAIL is reported to heighten the production of other pro-tumor inflammatory cytokines, such as IL-10, which inhibits the activity of NK cells and CD8+ T cells within the tumor microenvironment [47]. TRAIL can also enhance the secretion of matrix metalloproteinases (MMPs), including MMP2, MMP3, and MMP9, which promotes the epithelial-mesenchymal transition of esophageal squamous cell carcinoma cells [60]. Thus, cancer cells gain benefits from TRAIL signaling by secreting cytokines or chemokines, which may be a potential cause of TRAIL resistance.

### 3.4. TRAIL Modulates Cancer Angiogenesis

Angiogenesis, the formation of new blood vessels, occurs in normal physiological processes and in pathological conditions [61]. TRAIL could lead to vascular disruption by inducing apoptosis in DR5-expressing tumor endothelial cells and vascular smooth muscle cells, which effectively inhibits tumor growth [61,62]. However, TRAIL might also play an unexpected role in promoting angiogenesis. Studies have shown that soluble TRAIL could promote the migration of endothelial cells and the formation of new blood vessels to a degree comparable to vascular endothelial growth factor (VEGF) [63]. In breast cancer, endothelial cells at the premetastatic niche co-express TRAIL and DR5. The interaction between TRAIL and DR5 intracellularly stimulates endothelial cell proliferation, migration, and tubular formation, which potentially promotes angiogenesis and metastasis [64]. TRAIL could also promote angiogenesis by interacting with other factors, such as FGF-2 and NOX4 [65,66]. Hypoxia is a ubiquitous feature of the tumor microenvironment, and is able to inhibit TRAIL-induced apoptosis and stimulate angiogenesis [67]. Hypoxia triggers ROS production, which leads to increased oxidative stress. TRAIL has the ability to protect endothelial cells from oxidative stress by reducing ROS formation [68].

## 4. TRAIL as a Target for Cancer Gene Therapy

Since the discovery of TRAIL, its ability to specifically bind to trigger apoptosis in cancer cells has made TRAIL/DR a target for clinical tumor therapy [69]. Unlike conventional chemotherapy, which often leads to non-specific cytotoxic effects on healthy tissue, TRAIL’s selective action holds promise for reducing side effects and improving patient tolerance [70]. As a result, the development of TRAIL-based drugs has taken various directions and strategies.

Currently, TRAIL-based drugs are being evaluated in clinical trials for a variety of cancer types, including solid tumors like lung, breast, pancreatic, and colon cancers, as well as hematological malignancies such as lymphomas and advanced cancers (Table 1 and Table 2). However, none have advanced beyond phase III clinical trials. The development of TRAIL faces challenges related to its extremely short half-life, drug-resistant cancer cell populations, and inadequate in vivo delivery, which have hindered its clinical application [69,70]. Several labeled recombinant forms of TRAIL, despite their advantages in promoting ligand trimerization and enhancing agonist activity, have not been tested in clinical trials due to concerns about hepatotoxicity [3,71,72,73]. Subsequently, unlabeled recombinant soluble TRAIL (Dulanermin/AMG-951/APO2L) and Circularly permuted TRAIL (CPT) were introduced in clinical trials, but phase III clinical trials did not show improved overall survival (OS) in both experimental and placebo groups. In addition, severe responses were observed in phase II clinical trials [74,75]. Apo2L.XL, another human-engineered cross-linking form of Dulanermin, exhibits stronger pro-apoptotic activity, although its safety profile remains to be explored [76].

In clinical practice, antibody agonists targeting specific DR4 or DR5, effectively overcome the obstruction of TRAIL signaling by decoy receptors. These antibodies offer specific binding, high affinity, and longer half-lives [77]. Among various DR4 targeting antibodies, only the HGS-ETR1 antibody (Mapatumumab) has advanced to phase 2 clinical trials and demonstrated a good safety profile in several tumor therapies. However, its effectiveness has not been confirmed in other phase II clinical trials, and combining it with other drugs did not improve its efficacy [78,79]. Several clinical trials have been conducted with DR5 agonist antibodies, including Tigatuzumab (CS-1008) and conatumumab (AMG 655), which are currently in phase II clinical trials. Nevertheless, in vivo targeted therapies for several cancers have not achieved the desired results [74,80].

Studies have indicated that different cancer cells exhibit varying sensitivities to antibody agonists specific to DR4 or DR5 [81]. It has been suggested that apoptosis is induced under endoplasmic reticulum (ER) stress, and cell autonomous movement and invasion are described as occurring through TRAIL-R2. This suggests that targeting TRAIL-R1 for anticancer therapy may be more appropriate as it lacks pro-movement signaling [82].

In clinical studies, all agonistic TRAIL-R antibodies have been well tolerated, but the bivalent nature of monoclonal antibodies does not align with the mechanism of TRAIL trimer activation. This limits their ability to effectively cross-link TRAIL receptors and initiate apoptotic signaling. Innovative approaches such as the development of novel polyvalent antibodies aim to address this limitation, although cross-reactivity concerns remain to be resolved [80]. Novel DR5 polyvalent antibodies GEN1029, INBRX-109, and IGM-8444 have entered clinical trials. MEDI3039 is a novel multivalent DR5 agonist capable of inducing tumor regression in situ and inhibiting the growth of metastatic triple-negative breast cancer [83].

Innovative TRAIL therapies, including the development of more targeted and specific TRAIL derivatives, TRAIL couplings, and fusion proteins to overcome TRAIL resistance, require further clinical validation [74,84,85,86,87,88,89].

## 5. Novel Strategies for TRAIL-Based Therapy

**Figure 3:**
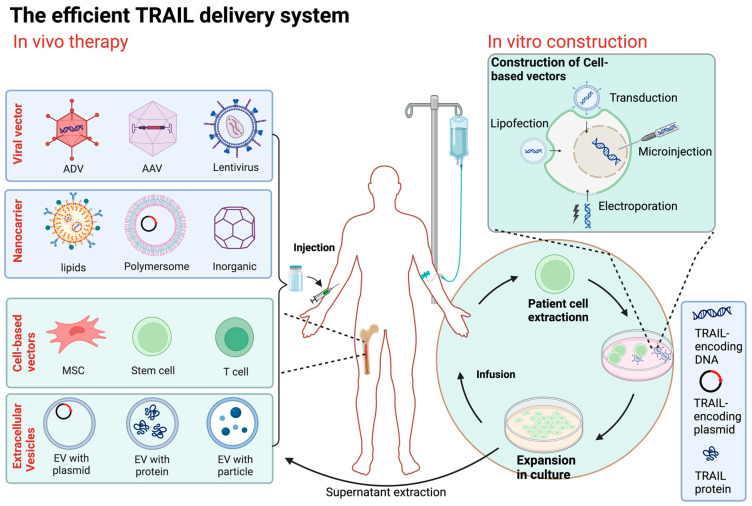
Overview of TRAIL delivery systems. The method of direct delivery is to inject the constructed TRAIL transgene vector (including viral vector and nanocarrier) directly into the patient. Cell carrier gene therapy involves obtaining cells from allogeneic or autologous sources, genetically modifying them with a carrier carrying the TRAIL gene or other physical means, selecting and expanding them in culture, and then injecting the engineered cells into the patient. ADV, adenovirus; AAV, adeno-associated virus; MSC, mesenchymal stem cell; EV, extracellular vesicle. (Figure was created with Biorender.com, accessed on 1 June 2024).

The journey of clinical development and application of TRAIL has been riddled with challenges and unexpected twists, particularly during the early stages. The primary objectives during this initial phase were to address the insufficient excitability of TRAIL/DR, its poor bioavailability, and the issue of TRAIL resistance. In recent years, a significant revelation has come to light—TRAIL exhibits a dual effect within the TME. This revelation necessitates a shift in focus towards enhancing the TME itself. In the subsequent discussion, we explore promising avenues for novel therapeutic strategies in the context of the TME. These strategies include combination therapy, specialized TRAIL delivery techniques, and other innovative approaches. These approaches take into account the pivotal role of the TME. Their primary aim is twofold: to enhance TRAIL’s bioavailability and its sustainability within the body, and to circumvent the development of drug resistance in tumor cells. Simultaneously, these strategies are designed to exert a regulatory influence on the TME, thereby creating a more conducive setting for TRAIL-based therapy (Figure 3).

In recent years, it has become clear that the TME plays a crucial role in the immunosuppression that shields tumor cells from immune cell attacks. To effectively control and treat cancer, it is essential to reverse this immunosuppressive environment. Several strategies aim to enhance TRAIL-induced apoptosis, focusing on targeting cells within the TME and improving its physical characteristics (Table 3).

### 5.1. TRAIL-Based Combination Therapy

#### 5.1.1. Combination with Chemotherapy or Radiotherapy

TRAIL receptor agonists (TRAs) combined with chemotherapy or radiotherapy have demonstrated higher efficacy in both preclinical and clinical trials than when used alone. The combination can effectively target TRAIL-resistant tumor cells, including tumor stem cells. The combination of TRAIL and doxorubicin, a combination that increases the expression of TRAIL receptors DR4 and DR5, promotes apoptosis and anti-angiogenesis in soft tissue sarcoma cells [90]. Additionally, first-line chemotherapy drugs like cisplatin and gemcitabine can sensitize tumor cells when used alongside TRAIL [91,92]. Other chemotherapy agents, including irinotecan, camptothecin, 5-FU, and platinum-based agents, have also been studied in combination with TRAIL [93]. These agents can induce endoplasmic reticulum stress, upregulate DR4 and DR5, and enhance TRAIL-induced apoptosis.

Radiation therapy, which causes DNA damage and stabilizes p53 levels, synergistically amplifies the effects of TRAIL on tumor cells. The experimental results showed that the combination of TRAIL and radiotherapy had an excellent therapeutic effect on cervical cancer cell lines [94]. While TRAIL can induce apoptosis independently of p53, the status of p53 can affect the sensitivity of tumor cells to TRAIL [92]. P53 can function as a pro-apoptotic component, similar to Bax, and also acts as a transcription factor for DR4 and DR5 expression [58].

Various drugs and biologics, such as proteasome inhibitors, Bcl-2 inhibitors, IAP antagonists, histone and deacetylase inhibitors, as well as natural compounds like resveratrol, quercetin, and Strophanthidin (SPTD), have been used in combination to sensitize tumor cells to TRAIL [95,96,97]. Overcoming drug resistance at different levels of the TRAIL signaling pathway presents challenges, and combination therapy emerges as a promising approach to address TRAIL resistance. However, it is crucial to carefully consider the choice of drug dosage and mode of administration to ensure the best synergies and effective antitumor effects. This includes precise dosing to the tumor site, co-dosing, and spatial dosing to enable more effective combination therapy.

#### 5.1.2. Combination with Immunotherapy

In recent years, the combination of TRAIL and immunotherapy has become more common as the immune role of TRAIL in the tumor microenvironment has been gradually revealed. One such strategy involves combining shTRAIL with the macrophage-targeting drug tribetidine, which can reshape the tumor immune microenvironment, bolster antitumor immunity, and significantly enhance the antitumor effects [47]. Additionally, combining mAb13F3 with T-cell-activated V-domain immunoglobulin suppressor (VISTA) and Toll-like receptor 3 (TLR3) specific adjuvant can upregulate TRAIL, increase the CD8+ T cell/Treg ratio in the TME, and induce macrophage activation [98]. Furthermore, MDSCs hinder CAR-T cell function and persistence within the breast TME. However, CAR-T cells equipped with chimeric co-stimulatory receptors targeting tumor-associated MUC1 (MUC1) can target DR5 expressed on MDSCs, enhancing antitumor potential and promoting TME remodeling and T cell proliferation at the tumor site [99]. Combining TRAIL with PD1 inhibitors can effectively block the PD1/PDL1 checkpoint signals, reactivating impotent tumor-specific CD8+ T cells and initiating an immune response through immunogenic cancer cell death [100]. In fact, combining TRAIL with other immune-related inhibitors has shown promise, especially when used alongside cytotoxic T-lymphocyte-associated protein 4 (CTLA-4) inhibitors and anti-programmed cell death protein 1 (PD-1) drug preparations (ICIs), which have reduced recurrence rates and improved survival rates to a certain extent.

#### 5.1.3. Combination with Microenvironment-Modulating Agents

Abnormalities in tumor blood vessels can result in inadequate blood supply and lead to a TME characterized by low glucose concentrations, low extracellular pH, and low oxygen levels. Improving the physical aspects of the TME can enhance TRAIL-induced apoptosis. Glucose deprivation, for instance, induces metabolic stress and promotes TRAIL-induced cytotoxicity. The combination of TRAIL and hypoglycemia has been shown to induce dose-dependent apoptosis by activating caspase (−8, −9, and −3). Glucose deprivation can also amplify BID-Bax-associated mitochondria-dependent pathways and enhance TRAIL-induced apoptosis [101]. Tumor cells upregulate various proteins involved in pH regulation, leading to an acidic extracellular TME that suppresses immune function and hinders the antitumor immune response. The use of pH-regulating drugs like Cariporide, Lansoprazole, and acetazolamide can partially alleviate this immune cell suppression. Cariporide treatment has the added benefit of reducing immunotherapy-associated CRC cell shedding DR5, DcR1, and PD-L1 [102]. For pancreatic ductal adenocarcinoma (PDAC), which has a rich desmosomal proliferative matrix, TRAIL delivery is hindered and tumor penetration is reduced. Nanotherapy of TRAIL not only removes the extracellular matrix barrier to increase TRAIL delivery to tumors, but also blocks anti-apoptotic mechanisms to overcome TRAIL resistance in PDAC. Co-delivery of TRAIL and NO via stroma-targeted nanogels can remodel the fibrotic TME and inhibit tumor growth, offering promising therapeutic potential for PDAC [103].

### 5.2. The Improved Efficient TRAIL Delivery System

Recombinant TRAIL proteins and antibodies targeting TRAIL receptors often face challenges in terms of rapid degradation and clearance from the systemic circulation due to their short half-life. This leads to insufficient exposure of tumors to TRAIL within the TME [46]. While increasing the frequency of TRAIL administration might seem like a solution to maintaining higher concentrations at the tumor site, this approach carries the risk of amplifying resistance and potential toxicity. Furthermore, many primary cancers exhibit intrinsic TRAIL resistance or acquire resistance after TRAIL treatment [3]. One of the fundamental hurdles in advancing TRAIL-based oncology therapy is the critical need for safe and effective delivery methods. Fortunately, recent advancements in viral vector, nanocarrier, and cellular vector delivery systems hold promise in overcoming the barriers that have hindered successful TRAIL administration in clinical trials. TRAIL-based delivery therapy offers the potential to enhance stability, prolong the plasma half-life of TRAIL, enable specific targeting, improve the TME, and overcome TRAIL resistance. These innovative delivery methods have the potential to revolutionize TRAIL-based cancer treatment (Figure 3).

#### 5.2.1. Viral Vector-Based TRAIL Therapy

For the high delivery efficiency, viral vectors, such as adenoviruses, adeno-associated viruses (AAVs), and lentiviruses, are prominently used for tumor-targeted gene delivery. TRAIL-based gene therapy is a highly promising approach for cancer treatment [4]. These vectors can integrate TRAIL expression genes into the genome of cancer cells, which enables selective TRAIL expression and efficiently induces cell apoptosis [104,105]. TRAIL-based gene therapy could also create a “bystander” effect, by which cell apoptosis could be induced in not only TRAIL-integrated cancer cells, but also the surrounding cells by the secretion of TRAIL proteins [106]. This characteristic enhances the therapeutic effect of TRAIL gene therapy.

Oncolytic adenoviruses (OAs) could selectively replicate within and kill cancer cells, which also enhances the antitumor effect of TRAIL [107]. Oncolytic viruses can also induce the host antitumor immune response to further mediate the destruction of tumor cells [108]. OA carrying dual therapeutic genes ST13 and TRAIL has proved to be safe and efficient treatment of pancreatic ductal adenocarcinoma [109]. In addition, the combination of luteolin and the OA carrying the TRAIL gene exerts synergistic antitumor effects in vitro and in vivo [110]. Recent studies have explored plant virus-based platforms, such as Potato virus X (PVX), for TRAIL delivery, showing enhanced efficacy in activating apoptosis [111]. These plant virus-based vectors can be combined with other treatments, such as doxorubicin, to further enhance the eradication of cancer cells [112]. Viral vectors can also carry immunomodulatory genes to stimulate an antitumor immune response. This approach has shown promise in the treatment of various cancers, such as malignant pleural mesothelioma and bladder cancer [113,114].

While the promise of TRAIL gene therapy using viral vectors is substantial, challenges and caveats exist. Viral vectors may have limitations in their ability to target specific cell types within tumors and can infect normal cells. Immune responses to viral vectors in the TME can limit their effectiveness, and strategies to mitigate immunogenicity are crucial for success [115]. Additionally, there is a risk of integration site insertion mutations, which can lead to unexpected genetic changes. It is crucial to identify the least genotoxic viral vector.

#### 5.2.2. Nanodelivery Systems

Nanoparticle materials used as nanocarriers have proven highly effective in transporting DNA encoding TRAIL into tumor cells, facilitating the local secretion of TRAIL proteins in the cell membrane or TME [116,117,118].

There are three main forms of TRAIL–nanoparticle assembly. The first is that TRAIL attaches to the surface of the NP, binding to TRAIL death receptors on tumor cells. The second is to encapsulate soluble TRAIL or TRAIL-encoding plasmids with nanoparticles (NPs). The last is a combination of the first two. Studies have shown that the pattern of TRAIL surface presentation has a better effect on TRAIL receptor aggregation and subsequent apoptosis induction than that of soluble TRAIL. For example, in sarcoma therapy, TRAIL-modified lipid nanoparticles have demonstrated greater cytotoxicity than soluble recombinant TRAIL [119]. Liposomes, polymers, and inorganic materials have been used to formulate TRAIL–nanoparticles (NPs) [117]. Liposome nanocarriers, in particular, serve as a common and important TRAIL delivery vector, significantly improving the TME and enhancing TRAIL’s apoptotic function. Research has shown that TRAIL-loaded liposomes effectively target and kill prostate circulating tumor cells (CTCs) in primary patient blood samples [120] and may systemically neutralize distant metastasis of a broad range of tumor types. Targeted delivery of TRAIL and the chemotherapy drug paclitaxel through nanocarriers has demonstrated success in enhancing remission in cases of melanoma drug resistance [121]. Multifunctional nanocarriers capable of co-loading human TRAIL coding plasmid genes and doxorubicin have been developed, showing promise in cancer therapy [122].

Nanovesicles carrying plasmids encoding TRAIL and arginine-coupled tocopherol lipids (ATS) offer a potentially safe and effective therapeutic strategy for gene therapy in glioblastoma [123]. Nanocarriers also facilitate the delivery of receptor agonist antibodies, such as DR5 antibodies conjugated with solid lipid nanoparticles for the selective targeting of cancer cells in triple-negative breast cancer (TNBC) therapy [124].

In addition, polymer and inorganic materials have their own advantages in TRAIL delivery. Wrapping DNA in NPs is a particularly attractive way to both prevent endonuclease degradation and extend the circulating half-life of DNA. In the context of TRAIL gene therapy, several polymer-based approaches, including polyethylenimide (PEI) and cationic dendrimers, have been used for in vivo delivery. However, the toxicity of high molecular weight PEI and the non-degradability of dendritic macromolecules are the factors that must be considered in TRAIL therapy [117]. In recent years, the development of inorganic nanomaterials has optimized the treatment of TRAIL. Graphene nanocarriers, when combined with DR4 antibodies and AKT siRNA, synergistically promote death receptor-mediated apoptosis and improve the therapeutic effect of tumors in vivo [125]. High TRAIL loading anodized alumina nanotubes (AANT) have exhibited excellent biocompatibility in breast cancer cells and immune response cells, inducing significant cell death within a very short latency period [126].

The combination of nanocarriers and viruses shows promise in cancer treatment. This approach addresses the challenges associated with viral gene therapy, where embedding adenovirus in nanoparticles or using nanoparticle formulations can enhance virus-mediated gene delivery, mitigate immunogenicity, and improve the safety and efficacy of viral vectors. Nanoparticles are also being explored in combination therapies, such as combining chemotherapy drugs with nucleic acid nanocarriers, to provide comprehensive cancer treatment [127].

#### 5.2.3. Cell-Based Vectors

Various cell vectors, including red blood cells (RBCs), white blood cells, macrophages, DC s, and stem cells, have been extensively employed as therapeutic drug carriers [128]. Engineered stem cells as TRAIL gene vectors show great therapeutic promise because of their role in improving the tumor microenvironment. Stem cell adipose-derived stem cells (ADSCs), modified with TRAIL, can migrate to hepatocellular carcinoma (HCC) cells, effectively inhibiting tumor growth and blocking the metastasis of transplanted HCC tumors [129].

However, it is mesenchymal stem cells (MSCs) that have been most extensively studied. Engineered MSCs have emerged as promising cancer therapy options due to their unique properties, such as low immunogenicity, high gene transduction capacity, and the ability to migrate to tumor sites [130]. Engineered MSCs, serving as gene carriers, can infiltrate tumor tissues and expand the potential scope of cancer treatment [131]. For pancreatic cancer treatment, TRAIL gene therapy based on bone marrow mesenchymal stem cells is a potential approach [132,133]. Additionally, photochemical internalization (PCI) has shown promising results in improving the transfection efficiency of TRAIL-secreting human mesenchymal stem cells (hMSCs) for pancreatic cancer treatment [133]. Mesenchymal stem cell-based cancer gene therapy can incorporate gene segments, such as Caspase9-mediated suicide genes, to enhance therapeutic potential [134]. In mouse lymphoma models, lentivirus-mediated expression of soluble TRAIL and IL-12 in transgenic MSCs effectively inhibits tumor growth and metastasis [135]. MSCs play various roles in the TME. TRAIL-expressing exosomes from MSCs exhibit potent antitumor activity, especially in mouse melanoma models. MSCs not only express TRAIL but also encapsulate a range of cytokines, chemokines, and growth factors in their secreted microvesicles and extracellular vesicles [136]. These biologically active entities are crucial for regulating cell growth, angiogenesis, and inflammation processes [137].

MSCs, in combination with various approaches, have demonstrated therapeutic efficacy, including combined radiotherapy, chemotherapy drugs, cytokines, histone deacetylase inhibitors (HDACi), and viral vectors [138,139]. For instance, in preclinical studies of pancreatic adenocarcinoma, nanocarriers carrying soluble TRAIL and paclitaxel delivered by mesenchymal stem cells have shown promising results [140]. The combined effect of umbilical cord-derived MSCs with IFN-γ and TNF-α enhances the treatment of acute myeloid leukemia [141]. The efficacy of MSC-based sTRAIL gene therapy combined with the histone deacetylase inhibitor panobinostat has been enhanced in the treatment of malignant glioma [142]. The identification of small molecule inhibitors that jointly target cancer stem cells (CSCs) and tumor-specific signaling pathways can enhance MSC-TRAIL-mediated tumor inhibition [143]. Using adenovirus-associated virus 2 (AAV2) as a vector, mesenchymal stem cells (MSCs) can be engineered to express CRC-specific sTRAIL [138]

#### 5.2.4. Cell Membrane/Extracellular Vesicle-Based Vectors

TRAIL binds to the surface of immune cells and uses its innate ability to transport throughout the cycle, avoiding clearance and uptake by the immune system while performing specific functions, including delivering nutrients to tissues, removing pathogens, and immune system surveillance.

Immune cells presenting TRAIL and the adhesion receptor E-selectin on their surface are employed to target and eliminate circulating colon and prostate cancer cells [144]. Functionalized natural killer (NK) cells with TRAIL and anti-NK1.1 combined liposomes target tumor metastasis in tumor-draining lymph nodes [145].

During the development of stem cell-based TRAIL cell vectors, extracellular vesicle-encapsulated TRAIL was also found to play an unexpected role in tumor therapy. Membrane vesicles (CIMVs) isolated from genetically modified human adipose tissue-derived mesenchymal stem cells (hADSCs) overexpressing TRAIL, PTEN, and IFN-β1 genes have immunomodulatory and antitumor properties. They can activate human immune cells in vitro and induce apoptosis in various types of cancer cells [146]. Induction of cytorelaxin B in MSCs leads to increased expression of TRAIL, PTEN, and IFN-β1 in vesicles, effectively eradicating malignant tumor cells [146]. Extracellular vesicles derived from mesenchymal stem cells protect against DISC (death-inducing signaling complex) downregulation through a microRNA-217-dependent mechanism [137]. Continuous expression and release of soluble TRAIL by umbilical cord mesenchymal stem cells (MSC-sTRAIL) effectively inhibit the proliferation and induce apoptosis of B-ALL (B-cell acute lymphoblastic leukemia) cells [147].

Exosomes from γdelta-T cells (γdelta-t-exos) maintain their tumor-killing and T-cell-promoting activity in the immunosuppressed nasopharyngeal carcinoma (NPC) microenvironment via the DR5/TRAIL pathway. γdelta-t-exos, in collaboration with radiotherapy, overcome the radiation resistance of nasopharyngeal carcinoma csc, effectively controlling nasopharyngeal carcinoma [148].

## 6. Conclusions

In the realm of modern medicine, the fight against cancer is an ongoing, formidable challenge that continually calls for innovative approaches. Among the many players in the intricate landscape of cancer biology, TRAIL has captured the attention of researchers and clinicians. TRAIL’s multifaceted role in cancer, particularly its involvement in shaping the TME, has become a focus of intense investigation. This article has delved into the complexities of TRAIL in cancer, shedding light on its diverse functions. TRAIL, we have discovered, is a double-edged sword, capable of both thwarting and, paradoxically, advancing cancer. It influences processes ranging from apoptosis to immune response modulation, playing a pivotal role in tumor initiation, progression, and invasion. Looking forward, the potential of TRAIL as a game-changer in cancer treatment is becoming increasingly evident. Innovative therapeutic strategies are emerging as promising avenues. Combination therapies, immunotherapies, and gene delivery techniques are being explored to harness the capabilities of TRAIL for the benefit of cancer patients. Furthermore, an ever-deepening understanding of the TME and its impact on TRAIL’s efficacy is paving the way for more precise and effective treatments. Finally, clinical differences in patient sensitivity to TRAIL have been found, and molecular typing of cancers is an aspect of TRAIL application that still needs to be explored, which may provide an important foundation for the future development of personalized TRAIL pathway-targeted therapies.

## Figures and Tables

**Table 1:** Clinical trials targeting TRAIL and its receptors.

Type	Drugs	Cancers	Phases	Clinical Trial ID
Rh TRAIL	Dulanermin (AMG 951)	NSCLC	II (2006–2011)	NCT00508625
		NSCLC	III (2016–2018)	NCT03083743
		Colorectal cancer	I (2009–2014)	NCT00873756
		NHL	I/II (2006–2010)	NCT00400764
		Colorectal cancer	I (2006–2012)	NCT00671372
	SCB-313	Peritoneal carcinomatosis	I (2019–2022)	NCT04047771
		Peritoneal malignancy	I (2018–2021)	NCT03443674
		Malignant pleural effusion	I (2019–2021)	NCT03869697
		Malignant ascites	I (2019–2022)	NCT04051112
		Malignant pleural effusion	I (2020–2022)	NCT04123886
TRAIL-R1 mAb	Mapatumumab (TRM-1/HGS-ETR1)	NHL	II (2004–2007)	NCT00094848
		NSCLC	II (2005)	NCT00092924
		Advanced cervical cancer	I/II (2010–2014)	NCT01088347
		HCC	I/II (2011–2017)	NCT01258608
		Multiple myeloma	II (2006–2010)	NCT00315757
TRAIL-R2 mAb	Tigatuzumab (CS-1008)	Pancreatic cancer	II (2007–2010)	NCT00521404
		Solid malignancy and lymphoma	I (2007)	NCT00320827
		TNBC	II (2011–2017)	NCT01307891
	Conatumumab (AMG 655)	Lymphoma	Ⅰ (2008–2011)	NCT00791011
		Colorectal carcinoma	II (2007–2011)	NCT00625651
		Colorectal carcinoma	II (2009–2012)	NCT00813605
		Unresectable soft tissue sarcoma	II (2007–2011)	NCT00626704
		Solid tumor	II (2009–2011)	NCT00819169
		Metastatic pancreatic cancer	I/II (2007–2012)	NCT00630552
	GEN1029	Malignant solid tumor	II (2018–2021)	NCT03576131
	INBRX-109	Chondrosarcoma	II (2021–2025)	NCT04950075
		Solid tumors including sarcoma	I (2018–2026)	NCT03715933
	IGM-8444	All-comers solid tumors	I (2020–2027)	NCT04553692
	Oba01(RC248-C001)	DR5 positive LA/mNSCLC	I (2023–2026)	NCT06083870
	DS-8273a	Advanced colorectal cancer	I (2016–2017)	NCT02991196
TRA	ABBV-621	Solid or hematologic malignancy	I (2017–2022)	NCT03082209

HCC, hepatocellular carcinoma; NHL, Non-Hodgkin’s lymphoma; TRA, TRAIL receptor agonist.

**Table 2:** Clinical trials targeting TRAIL signaling pathways.

Type	Drugs	Cancers	Phases	Clinical Trial ID
IAPs inhibitor	Xevinapant	SCCHN	III (2023–2030)	NCT05930938
		SCCHN	II (2024–2028)	NCT06084845
Bcl-2 inhibitor	Venetoclax (ABT-199/GDC-0199)	CLL	I/II (2015–2024)	NCT02427451
		Solid malignancy	II (2018–2023)	NCT03552692
		Breast cancer	I (2019–2025)	NCT03900884
	ABT-263 (Navitoclax)	HGSC and TNBC	I (2022–2025)	NCT05358639
		CLL	II (2010–2012)	NCT01087151
	AVALON	AML	I (2019–2020)	NCT04070807
	BGB-11417	Mature B-cell malignancy	I (2020–2027)	NCT04277637
	BGB-21447	Mature B-cell malignancy	I (2023–2026)	NCT05828589
	TQB3909	Breast cancer	I/II (2023)	NCT05775575
		Malignancy	I (2022–2024)	NCT04975204
	LOXO-338	Blood cancer	I (2021–2023)	NCT05024045
	ZN-d5	AML	I (2022–2026)	NCT05682170
	APG-2575	SCLC	I (2017–2024)	NCT03387332
	FCN-338	CLL	I (2021–2024)	NCT04682808
Bcl-2 DNAi	PNT2258	Diffuse large B-cell lymphoma	II (2014–2016)	NCT02226965
L-Bcl-2	Oblimersen	WM	I/II (2003–2007)	NCT00062244
		Solid malignancy	I (2001–2010)	NCT00636545
	BP1002	AML	I (2022–2024)	NCT05190471
	G3139	SCLC	I/II (2000–2001)	NCT00005032
		Solid malignancy	I (2005–2006)	NCT00543231
		RCC	II (2003–2005)	NCT00059813
Bcl-XL Inhibitor	Bcl-XL_42-CAF09b Vaccination	Prostate cancer with lymph node metastases	I (2018–2021)	NCT03412786
	AT-101	Laryngeal cancer	II (2012–2021)	NCT01633541
Bcl-2 family Inhibitor	Pelcitoclax (APG-1252)	Neuroendocrine tumor	I (2022–2025)	NCT04893759
		SCLC	I (2017–2021)	NCT03387332
	Navitoclax	HGSC and TNBC	I (2022–2025)	NCT05358639
Bcl-2 and MCL-1 Inhibitor	VOB560-MIK665	NHL, MM and AML	I (2021–2024)	NCT04702425
MCL-1 Inhibitor	ABBV-467	MM	I (2020–2021)	NCT04178902
	Murizatoclax (AMG 397)	Hematological malignancy	I (2018–2019)	NCT03465540
	PRT1419	Relapsed or refractory myeloid	I (2022–2024)	NCT05107856
	MIK665 (S64315)	MM	I (2017–2019)	NCT02992483
		AML and MDS	I (2017–2020)	NCT02979366
		AML	II (2021–2024)	NCT03672695

AML, acute myeloid leukemia; Bcl-2 family, Bcl-XL, Bcl-2 and Bcl-w; L-Bcl-2, Bcl-2 antisense oligonucleotide; Bcl-2 DNAi, Bcl-2 targeted deoxyribonucleic acid inhibitor; BPDCN, blastic plasmacytoid dendritic cell neoplasm; CLL, chronic lymphocytic leukemia; HGSC, ovarian high grade serous carcinoma; MCL, mantle cell lymphoma; MDS, myelodysplastic syndrome; MM, multiple myeloma; NHL, Non-Hodgkin lymphoma; RCC, renal cell cancer; SCCHN, squamous cell carcinoma of the head and neck; SCLC, small cell lung cancer; WM, Waldenstrom’s macroglobulinemia; TNBC, triple-negative breast cancer.

**Table 3:** Clinical trials targeting the TRAIL signal in the immune microenvironment.

Cancers	Drugs	Phases	Outcome	Clinical Trial ID
Metastatic Pancreatic Cancer	AMG 655 or AMG 479 targets DR5 on MDSCs	I/II (2007–2012)	Completed	NCT00630552
Metastatic Renal Cancer	The α and β signaling chains of 2G-1 transduced into human lymphocytes by retroviral vectors	I (2009–2012)	Terminated	NCT00923390
Unresectable Stage III or Stage IV melanoma	DS-8273a combined with Nivolumab (anti-PD-1 antibody)	I (2016–2021)	Completed	NCT02983006
Solid Malignancies	Autologous CAR-T/TCR-T Cell combined with anti-DR5 antibody	I (2019–2021)	Completed	NCT03941626
Metastatic NSCLC	Targeted stem cells expressing TRAIL combined with pemetrexed/cisplatin chemotherapy	I/II (2019–2025)	Ongoing, recruiting	NCT03298763
Metastatic Breast Cancer	TRAIL-R2 and HER2 bi-specific CAR-T cells combined with IL-15	I (2024–2033)	Ongoing, recruiting	NCT06251544

2G-1, α and β signaling chain of cloned T-cell receptor; PD-1, programmed death-1; CAR-T cells, chimeric antigen receptor modified T cells; TCR, T-cell receptor.

## Data Availability

No new data were created or analyzed in this study. Data sharing is not applicable to this article.
